# Ultrafine Friction Grinding of Lignin for Development of Starch Biocomposite Films

**DOI:** 10.3390/polym13122024

**Published:** 2021-06-21

**Authors:** Seyedeh Najmeh Mousavi, Noureddin Nazarnezhad, Ghasem Asadpour, Sunil Kumar Ramamoorthy, Akram Zamani

**Affiliations:** 1 Department of Wood and Paper Science, Faculty of Natural Resources, Sari Agriculture Science and Natural Resources University, P.O. Box 578, Sari 4818168984, Iran; najmeh.mousavi@hb.se (S.N.M.); n.nazarnezhad@sanru.ac.ir (N.N.); gh.asadpour@sanru.ac.ir (G.A.); 2 Swedish Center for Resources Recovery, University of Borås, 50190 Borås, Sweden; sunil.kumar@hb.se

**Keywords:** soda black liquor, lignin, starch, biocomposite, ultrafine grinding

## Abstract

The work demonstrates the utilization of fractionalized lignin from the black liquor of soda pulping for the development of starch-lignin biocomposites. The effect of ultrafine friction grinding on lignin particle size and properties of the biocomposites was investigated. Microscopic analysis and membrane filtration confirmed the reduction of lignin particle sizes down to micro and nanoparticles during the grinding process. Field Emission Scanning Electron Microscopy confirmed the compatibility between lignin particles and starch in the composites. The composite films were characterized for chemical structure, ultraviolet blocking, mechanical, and thermal properties. Additional grinding steps led to the reduction of large lignin particles and the produced particles were uniform. The formation of 7.7 to 11.3% lignin nanoparticles was confirmed in the two steps of membrane filtration. The highest tensile strain of the biocomposite films were 5.09 MPa, which displays a 40% improvement compared to starch films. Further, thermal stability of the composite films was better than that of starch films. The results from ultraviolet transmission showed that the composite films could act as an ultraviolet barrier in packaging applications.

## 1. Introduction

The scientific community has been continuously looking for environmentally friendly materials to replace petroleum-based polymers. Starch is an abundantly available biopolymer found in many crops such as potatoes, rice, and wheat. The starch can be extruded as thermoplastic melt under high temperature and pressure after an addition of low levels of plasticizers such as water or glycerol. This thermoplastic melt or thermoplastic starch is renewable and flexible, and can be used to produce polymer films. Nevertheless, the use of these films is limited due to poor mechanical properties and low resistance to humidity. Researchers applied several strategies such as polymer blends and the addition of reinforcements to overcome the limitations. Incorporation of wood dust to thermoplastic starch films has resulted in improvement of the tensile strength of the obtained biocomposite material [1].

Black liquor is a by-product of paper and pulp industry that contains 30–50% lignin in the form of alkali or sulfonated lignin. Soda and Kraft lignin include alkaline lignin. During the soda process, the lignocellulosic materials such as bagasse are subjected to a cooking process using sodium hydroxide, where the molecular weight of lignin is reduced and lignin is solubilized. This results in a paper pulp containing mainly cellulose as well as a black liquor rich in lignin [2,3]. The solubilized lignin can be recovered by precipitation at pH 2–3 followed by filtration [4].

With 50–70 million ton per year annual production, lignin is a very promising resource for diverse application such as concrete additives, binder, dispersants, lubricants, and cosmetics [5]. Moreover, the recovered lignin can be used as filler and reinforcement in starch based films. Starch based films are good candidates for engineering and packaging applications [5]. However, mechanical, hygroscopic, and barrier properties of starch films need to be improved using fillers and property enhancement agents [6]. Lignin has a highly complex aromatic heteropolymer network. This network contains phenylpropane units with highly cross-linked aromatic groups. This limits the molecular motion and increases the glass transition temperature (T_g_). This makes the lignin a good choice for reinforcement in packaging application [7].

Starch films have been reinforced with 3–10% of lignin recovered from softwood [8,9], hard wood [10,11], and wheat straw [12]. Starch lignin films are produced by mixing of an aqueous solution of starch with lignin solution using casting method [13]. Lignin-starch films showed improved ultraviolet protective and mechanical properties compared to the films made of starch [3,13,14]. However, higher lignin concentrations than 10% in starch films can negatively affect the mechanical properties of starch films [15]. Nonetheless, heterogeneous and non-uniform structures of lignin have limited its application as reinforcement in starch films [4].

Reduction of lignin particle size would allow lignin to be an effective reinforcement and improve the properties of the composite as the decreased particle size lead to an increase in the surface area for bonding [16]. The nanoparticle can provide a large surface area and more sites available for chemical modification [17] and consequently can improve mechanical and barrier properties, and thermal stability [18]. There are several chemical methods to prepare nanolignin, such as chemical treatments including anti-solvent precipitation [19], acid precipitation [20] and self-assembly [21]. However, these methods can be in contrast to the environment-friendly processes, as harsh solvents or chemicals are used in these processes [22]. Furthermore, the attempt to produce nanoparticles of lignin by mechanical methods such as ultrasonication [23], and high shear homogenizer [24] are developing topics. Among mechanical treatments, the ultrafine friction grinding process using a grinder called “supermasscolloider” can result in fine and round-shape particles. The process has been used for the production of ultrafine powders of, e.g., food, chemicals, and medicine [25,26]. The process has also been used for fibrillation of cellulose and formation of nanocellulose and nanolignocellulose [27,28,29,30]. However, ultrafine friction grinding has never been used for size reduction of lignin and development of micro and nanoparticles.

The primary objective of this study was to examine ultrafine friction grinding process for size reduction of lignin recovered from soda black liquor. The second goal was to use the ground lignin as a filler in starch based biocomposites. The effect of the lignin particle size on mechanical, thermal, and ultraviolet protection properties of the films was investigated. Improvement in mechanical, thermal, and ultraviolet blocking properties could lead to the use of biocomposite for packaging applications.

## 2. Materials and Methods

### 2.1. Materials and Chemicals

Soda black liquor of sugarcane bagasse was received from the Pars paper mill (Khuzestan, Iran). Total solids, total lignin, Klason lignin, mineral element and organic extractive were 47.7, 38.3, 28.0, 24.0, and 20.2 wt%, respectively. Wheat starch was purchased from Sigma Aldrich (Stockholm, Sweden) and was used in experiments as supplied. Sulfuric acid (97%) supplied by Scharlau (Madrid, Spain), sodium hydroxide (98%) supplied by Sigma Aldrich (Stockholm, Sweden), and glycerol supplied by VWR Chemicals BDH (Leverantor, Belgium) were also used in the experiments.

### 2.2. Lignin Extraction

Lignin was precipitated by acidification of soda black liquor using a procedure used by Zhu et al. [31]. Black liquor was heated for 1 h at 80 °C in a water bath (Memmert, Model WNB 22, Schwabach, Germany) while it was manually mixed every 10 min. Then, the pH of the liquor was adjusted to 2–3 by adding 6 M sulfuric acid, under mechanical stirring (IKA, RCT B S000, Staufen, Germany). The liquor was returned to water bath at 80 °C to complete precipitation. After 1 h, the liquor was vacuum filtered using filter paper with 2.5 µm pore size (Whatman, Track-Etch membrane, New Jersey, USA) to separate the precipitated lignin. Lignin was washed with hot water 3 times, until pH of the filtrate was 6–7. Finally, lignin was dried at room temperature and kept in plastic bags until use.

### 2.3. Ultrafine Grinding of Lignin

A disk mill grinder called supermasscolloider (MKCA6-5J, Masuko Sangyo, Kawaguchi, Japan) was used for grinding of lignin. A suspension of lignin in water (2%) was prepared and the pH was adjusted to 7. The pH adjustment was done to improve nanoparticle stability according to the previous studies [22,32]. Two liters of lignin suspension were subjected to different cycles of grinding up to 100 times in a contact mode for the grinding stones. The first 40 cycles were performed using MKE46 grinding stones that are fine silicon carbide standard grinding stones for soft materials while the rest of grinding cycles were done using MKGA/GC 80 grinding stones, which are ultrafine silicon carbide stones for pulverizing and emulsifying fibrous materials. The rotation speed of the stones was 1500 rpm and the distance between the stones was gradually adjusted to −70 µm (contact mode). Samples were taken from the lignin suspension after 10, 20, 30, 40, and 100 grinding cycles. To reduce aggregation of lignin particles, the collected samples were kept in a water bath shaker (Shaking water bath, model OLS200, Cambridge, UK) at 25 °C and mixed at 100 rpm until use.

### 2.4. FluidScope^TM^ Scanning Technology (oCelloScope)

The automated time lapse microscopy pictures recorded with the FluidScope^TM^ scanning technology with the oCelloScope (BioSense Solutions ApS, Farum, Denmark) were used to determine the size distribution of lignin micro particles after grinding. Lignin suspensions in water were diluted 500 times before imaging. Images were captured from 3 samples of each lignin suspension, obtained at different grinding cycles and the number of images for each sample was set to 20. EQPC algorithm provided by UniExplorer software was used to illustrate lignin particle size.

### 2.5. Membrane Filtration

To determine the fraction of lignin nanoparticles created in the grinding process, the lignin suspension (obtained after 10, 20, 30, 40, and 100 grinding cycles) was diluted 20 times using water and passed through a membrane filtration unit (KOCH membrane system, Wilmington, US) with 100 nm pore filter (Whatman, Track-Etch membrane, Florham Park, NJ, USA) at 2 bar. The filter machine prevented the rapid formation of lignin cake on the surface of the membrane by internal flow circulation and thus allowed passage of lignin nanoparticles through the filter. The filtration was done in two steps. The liquid filtrate which passed the membrane (containing the nanoparticles) was collected and the lignin suspension that stayed on the top of the membrane (retained) was subjected to another filtration step using the same type of membrane. A sample (15 mL) of the filtrates obtained in each step was dried at 100 °C for 2 h to determine the concentration of the lignin nanoparticles in the filtrate.

### 2.6. Preparation of Starch-Lignin Films

A mixture of 5% starch in distilled water was heated to 90 °C for 30 min under 500 rpm stirring to dissolve the starch. Then, the starch solution was cooled to 60 °C, and lignin suspensions obtained at different grinding cycles were added to the starch solution. Based on preliminary experiments, the ratio of lignin/starch was chosen as 10 wt%. This concentration resulted in films with high mechanical properties compared to higher lignin concentrations at which proper distribution of lignin in starch matrix was not possible. Thirty percent glycerol, based on starch, was added as a plasticizer as suggested by Basiak et al. [33]. The mixture was mixed manually and cast in Teflon Petri dishes with 10 cm diameter and died at 45 °C for 24 h. For comparison, starch films were also produced without the addition of lignin. The films were stored in plastic bags at room temperature until use. Starch-Lignin films were marked as SLX, in which X refers to the number of lignin grinding.

### 2.7. Tensile Testing

Mechanical properties of the starch-lignin films were studied by tensile testing according to ISO 527-1996 using tensile tester (Tinius Olsen H10KT universal, Salfords, UK) equipped with QMat software. Films were cut to dog bone specimens with 8.5 cm length following the standard. A load cell of 100 N and a gauge length of 25 mm was used for the measurement. A crosshead speed of 10 mm/min without a preload was applied. From each film, at least three specimens were tested.

### 2.8. Thermal Analysis

To determine the thermal degradation behavior of starch-lignin films Thermogravimetric analysis (TGA) was conducted using Q500 TA instruments (Waters LLC, New Castle, DE, USA). Fifteen mg of the films were heated from room temperature to 700 °C at the rate of 10 °C/min under nitrogen atmosphere.

Differential scanning calorimetry (DSC) analysis was carried out using Q2000 TA Instruments (Waters LLC, New Castle, DE, USA) to study the effect of lignin on thermal stability of the starch-lignin films. Of the films, 6–7 mg was heated from −20 to 250 °C at the rate of 10 °C/min, and each experiment included 3 cycles of heating and cooling under nitrogen atmosphere.

### 2.9. FTIR Analysis

The FTIR spectra of lignin before and after grinding, as well as the spectra of the starch-lignin films were recorded using a Fourier transform infrared (FTIR) spectrometer (Thermo Scientific, Nicolet Is 10, Waltham, USA) in absorbance mode with 64 scans and a resolution of 4 cm^−1^. OMNIC software was used for data analysis.

### 2.10. UV Spectrophotometry

The transmittance of the films was measured in a light wavelength area between 200 and 800 nm by a UV—visible spectrophotometer (Biochrom libra S50, Cambridge, UK).

### 2.11. Optical Microscopy and Field Emission Scanning Electron Microscopy

The surface morphology of the produced films was captured from the surface of starch and starch-lignin films using an optical microscope (Nikon SMZ-800, Tokyo, Japan). Furthermore, field emission scanning electron microscope (FESEM) (Sigma, ZEISS, Oberkochen, Germany), was used to study the morphology of the films. Samples were attached to a carbon tape and covered with gold before imaging. Images were taken using an accelerating voltage of 20 kV.

### 2.12. Statistical Analysis

Error values and bars are presented with the standard deviation, unless indicated otherwise. The experiments were conducted in duplicate unless otherwise stated. Statistical analysis was performed using analysis of variance (ANOVA) and post hoc Tukey test. Significant differences were considered at *p*-value < 0.05.

## 3. Results and Discussion

Lignin-starch composites have been studied widely. However, the effect of lignin particle size on the properties of lignin-starch composite has scarcely been investigated. For this purpose, recovered lignin from black liquor was subjected to ultrafine grinding at different cycle times. Starch lignin films were produced and a comparison was done between pure starch film and the composite of ground lignin with starch.

### 3.1. Grinding of Lignin and Determination of Lignin Particle Size

**Figure 1:**
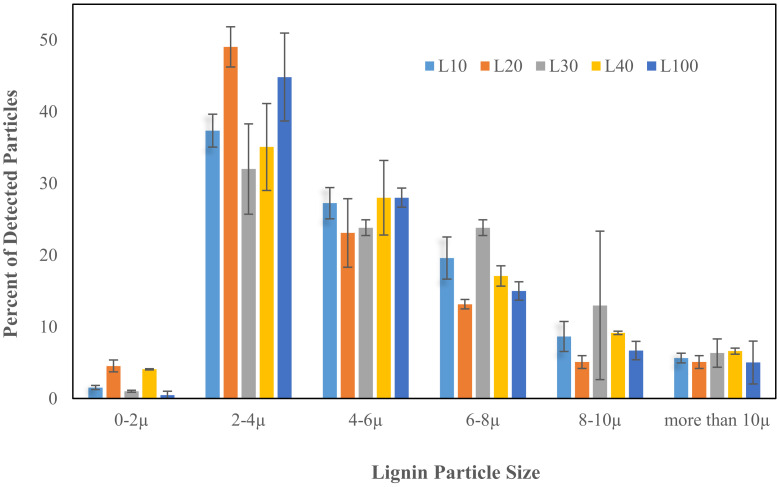
Distribution of lignin particle size after different grinding cycles, obtained by analysis of images, taken using oCelloScope and UniExplorer software.

**Figure 2:**
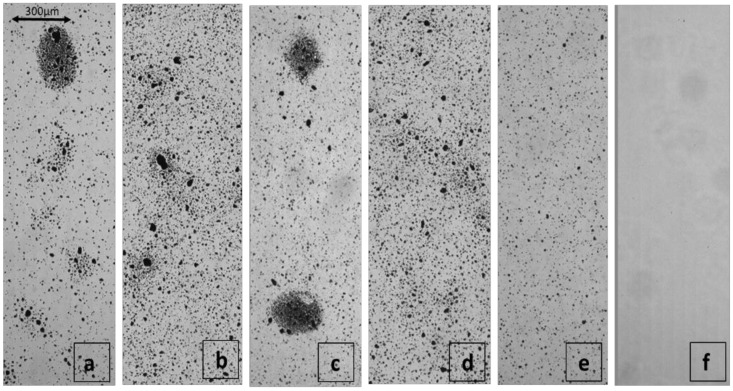
Microscopy images of lignin suspension in water obtained using oCelloScope after different grinding cycles of (**a**) 10, (**b**) 20, (**c**) 30, (**d**) 40, and (**e**) 100. (**f**) Represents the microscopic image of lignin nanoparticles suspension recovered as filtrate in the membrane filtration indicating no visible microscopic particles.

**Figure 3:**
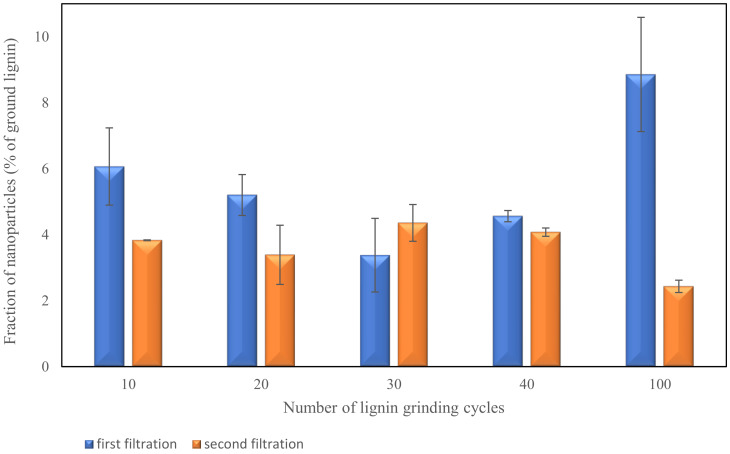
Fraction of lignin nanoparticles in ground lignin suspension measured in two filtration steps for different grinding cycles.

The morphology and distribution of lignin particle size after 10, 20, 30, 40, and 100 circulated steps of super grinding were recognized and analyzed by oCelloScope (Figure 1 and Figure 2). It classified the size of the particles between 0 and 2, 2 and 4, 4 and 6, 6 and 8, 8 and 10 and more than 10 micrometers, as shown in Figure 1. The presence of nanoparticles after grinding was confirmed by Nano membrane filtration, as illustrated in Figure 3. It was evident from these figures that the ultrafine grinding reduced lignin particle size. After 30 circulated steps, almost 96% of micro particles were under 10 micrometers. After 100 steps, the ratio of small to large micro particles was reduced. This is because the particles were ground to nanoscale, which was not detectable by oCelloScope anymore. Figure 2 shows the aggregation of lignin particles up to 30 grinding cycles as well as the uniform distribution of particles at the end of 100 grinding cycles.

Figure 2f was taken after membrane filtration, where no micro particles were observed in the filtrate, despite dark solids being observed after drying of this liquid. To the best of our knowledge, there have been no published studies on pure lignin grinding and this is the first time pure lignin particle size has been reduced to nanoscale using an ultrafine grinding process. However, Liu et al. [34] decreased the diameter of bagasse and Kraft cellulose fibers by ultrafine grinding until 23 and 25 nm, respectively. Reducing lignin particle size by ultrafine friction agreed with the values reported for cellulosic nanofibers by other researchers [25,27,28,35].

The samples from each step of lignin grinding were examined by membrane filtration to evaluate the effect of the number of grinding steps to produce lignin nanoparticle. Lignin nanoparticles were separated from the micro particles in step 2 of the filtration process. The high amount of solid nanoparticles was obtained (as measured by drying the filtrate) after the first filtration step while the second filtration step increased the yield slightly. Figure 3 shows the number of nanoparticles extracted in each filtration step for different grinding cycles. The total amount of Nano lignin after two filtration steps was about 7.7–9.9% for 10–40 grinding cycles. Increasing the number of grinding cycles to 100, resulted in a total nanoparticle content of 11.3% in the ground lignin suspension. However, statistical analysis showed no significant changes (*p*-value > 0.05) in the total amount of lignin nanoparticles detected by membrane filtration for different grinding cycles. The ground lignin suspensions were used directly for the production of starch lignin films.

### 3.2. Morphology of Starch and Lignin-Starch Composite

**Figure 4:**
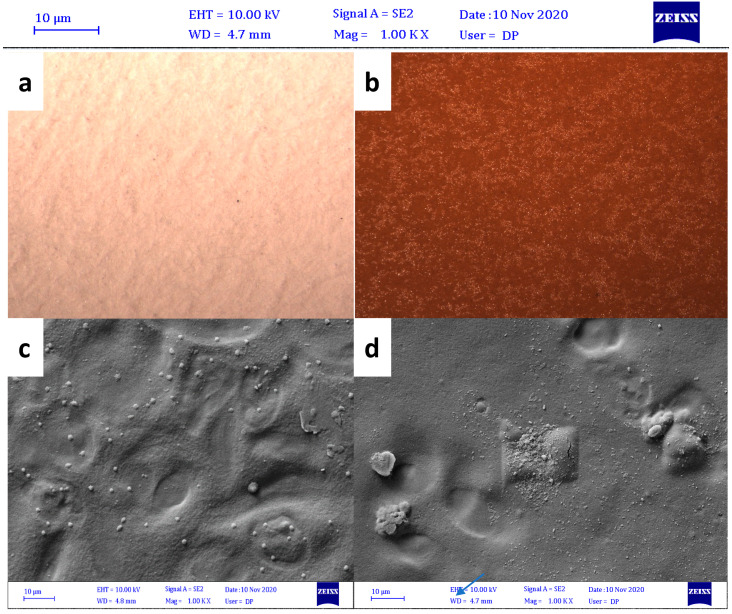
Optical Micrographs of (**a**) neat starch and (**b**) lignin-starch composite in 100 µm scale, (**c**) FESEM images of neat starch in 10 µm scale, and (**d**) FESEM images of starch—lignin composite containing 10 cycle ground lignin in 10 µm scale.

The optical micrographs of neat starch and starch-lignin composites are shown in Figure 4. Even though it does not explain the interaction between starch and lignin in nanoscale, it was effective in observing the distribution of lignin particles in the starch matrix in a wide area. Lignin composite appeared to be semitransparent light brown color compared to transparent film obtained from starch.

Figure 4c shows SEM images of pure starch. White micro particles in the pure starch points to few undissolved starch granules and glycerol penetration into it. Figure 4d shows the surface of the starch-lignin composite. In a qualitative study, the figure showed that the composites have only a few bubbles and pores on the surface. This indicates a good compatibility and interaction between micro and nanoparticles of lignin and starch. The images of starch composites containing lignin particles were documented in several studies and the results of this study fall in line with previous work (Miao et al. [36], Shi et al. [37], Espinoza Acosta et al. [38], and Spiridon et al. [39]).

### 3.3. Mechanical Properties

The mechanical performance of biopolymer films is commonly characterized by tensile strength and elongation at break. The tensile strength and elongation charts of neat starch and lignin-starch blends in different grinding cycles are shown in Table 1. To study the effect of grinding cycles, all the composites have 10% lignin content while the lignin comes from different grinding cycles. The tensile strength of the starch films increased by the addition of lignin particles from grinding cycles 10 to 40 while the strength decreased on the introduction of lignin from grinding cycle 100. The highest (40%) increase in the starch film’s tensile strength was achieved when lignin from 10 grinding cycles was used. Therefore, even though, higher grinding cycles result in smaller particles and higher surface area of ground lignin, this increases the risk of lignin agglomeration during composite production by casting. Agglomeration is a well-known issue while reinforcing of starch with lignin nanoparticles and this may lead to the uneven structure of lignin-starch films [40]. Elongation in all composite films was lower than that of neat starch films. This is due to the rigid construction of lignin, which causes the starch-lignin films to be stiffer than neat starch [9,41]. Similar observations in tensile strength and elongation at break of lignin-starch composites have been reported by Bhat et al. [42]; Zhang et al. [41]; Espinoza Acosta et al. [38], and Kaewtatip et al. [9]. Several studies suggest that the incorporation of high concentrations of lignin may negatively affect the mechanical properties of the composites [40,43,44].

In order to study the effect of grinding cycles, all the composites were made using 10% lignin content while the lignin comes from different grinding cycles. There was a significant difference between the tensile strength of the neat starch films and biocomposites (*p*-value = 0.0001). An addition of lignin particles from 10 grinding cycles increased the tensile significantly (*p*-value = 0.01). Tensile strength of biocomposite films made using L20, L30, and L40, was not significantly different from biocomposite made using L10 (*p*-value > 0.05). However, biocomposite made using SL100 had a significantly lower tensile compared to the one made using SL10 (*p*-value = 0.001). Therefore, the highest (40%) increase in the starch film’s tensile strength was achieved when lignin from 10 grinding cycles was used. Moreover, higher grinding cycles resulted in smaller particles and higher surface area of ground lignin, however, this increased the risk of lignin agglomeration during the course of composite production by casting.

Elongation in all composite films were lower than that of neat starch films. This is due to the rigid construction of lignin which cause the starch-lignin films to be stiffer than neat starch. There was a significant difference between the elongation at break of the starch film and biocomposite film made using L10 (*p* value = 0.016). However, there was no significant difference between the elongation at break of biocomposites made using lignin from different grinding cycles.

### 3.4. Thermal Properties

**Figure 5:**
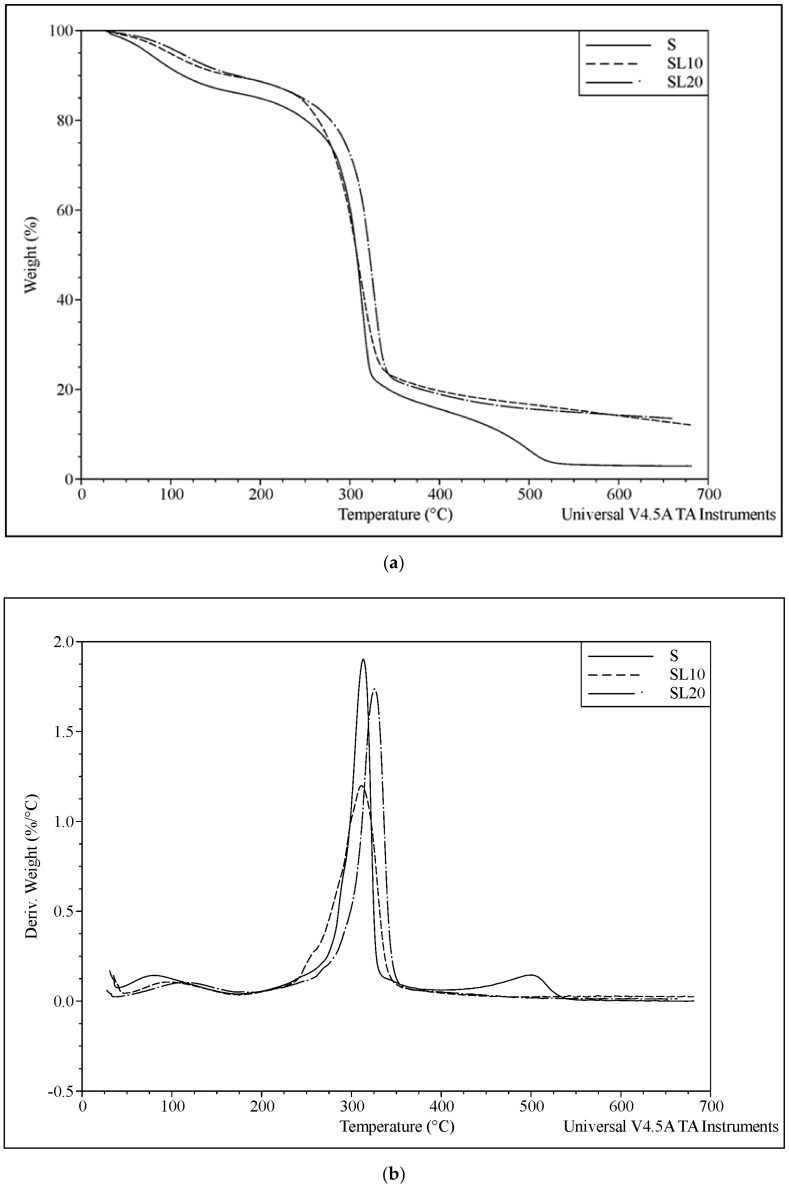
(**a**) Thermogravimetric analysis of neat starch (S) and starch-lignin composite (SL10, and SL20) films at different number of grinding cycles. (**b**) Derivative thermogravimetric analysis of neat starch (S) and starch-lignin composite (SL10, and SL20) at different number of grinding cycles.

Thermogravimetric analysis was used to follow the thermal degradation through weight loss of the films over a temperature range. Maximum degradation temperatures of starch composites and their derivatives were analyzed as well. Thermal stability of the starch films improved slightly with the addition of lignin, as shown in Figure 5a,b. This was significant below 250 °C and over 400 °C. Ten percent lignin content in the composite films was reflected in the residue at 650 °C, confirming the reinforcement addition to the starch. Maximum degradation of all composite samples occurred between 310 °C and 330 °C. Lignin decomposition is a complicated process because of various aromatic groups in the lignin structure. In Figure 5a, the first degradation around 100 to 150 °C is attributed to the volatilization of glycerol and water. The following steps include depolymerization of starch around 200 °C caused by amylose component [42] and lignin thermal degradation between 280 °C and 340 °C [14]. Lignin-starch composite shows higher thermal stability than neat starch because of the increased intermolecular hydrogen bonds between lignin and starch [43]. Thermal stability was affected by the number of grinding cycles. This could be due to increased hydrogen bonding between lignin and starch caused by a surface area increase through micro- and nanoparticle size of lignin. The improvement in the thermal stability of the starch films on lignin introduction falls in line with previous studies [9,38].

**Figure 6:**
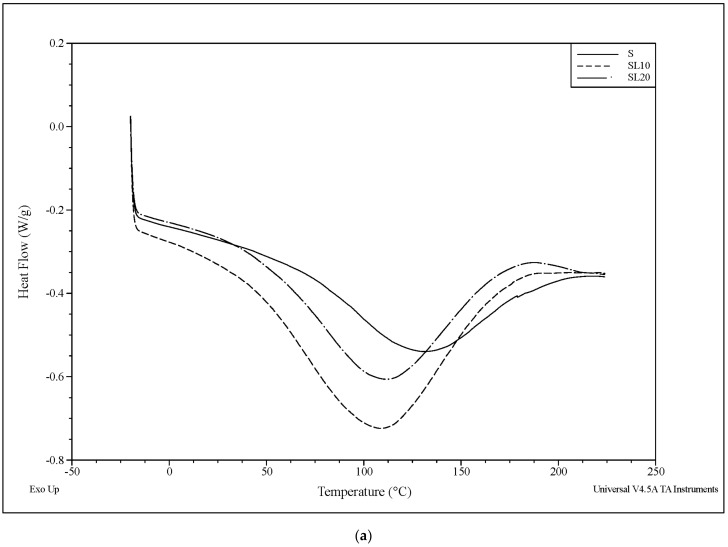

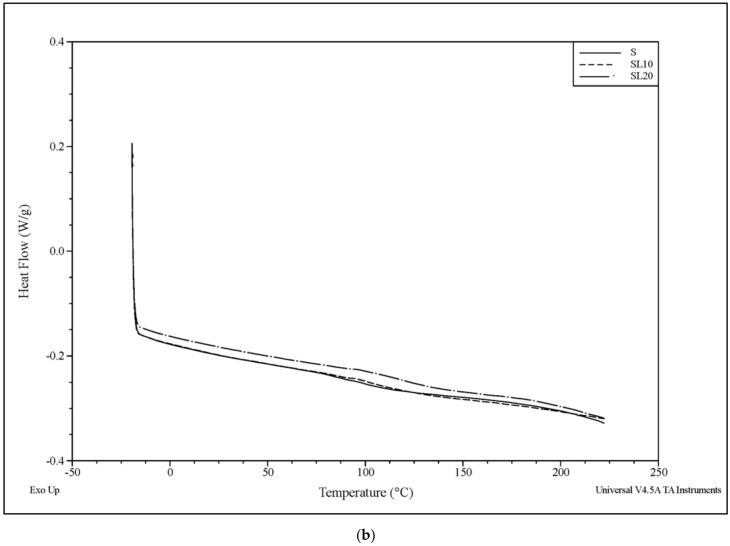
(**a**). Differential scanning calorimetry curves from first heating cycle of neat starch and starch–lignin composite with a different number of super grinding cycles. (**b**) Differential scanning calorimetry curves from second heating cycle of neat starch and starch–lignin composite with a different number of super grinding cycles.

DSC curves of starch films and composite films shown in Figure 6a,b. The peak noticed in the first heating cycle (Figure 6a) attributed to absorbed moisture disappears in the second heating cycle. In the Figure 6b, endothermic peaks were observed no longer but there was a change of inclination of baseline at 120° C, which corresponded to the T_g_ of the films. The absence of the endothermic peak films in the temperature region 100–200 °C indicated the amorphous nature of the films. Inclination of baseline observed for the composite films in this temperature region was due to the presence of lignin; indicating the molecular chain movements in lignin as the lignin’s glass transition temperature (T_g_) lies between 100 and 150 °C [44]. The number of grinding cycles affected the T_g_ slightly. T_g_ of plasticizers such as glycerol could also be affected but this transition located in the temperature region from −80 to −50 °C cannot be studied in this work due to the limitation of the DSC cooling system.

### 3.5. Ultraviolet Barrier Properties

**Figure 7:**
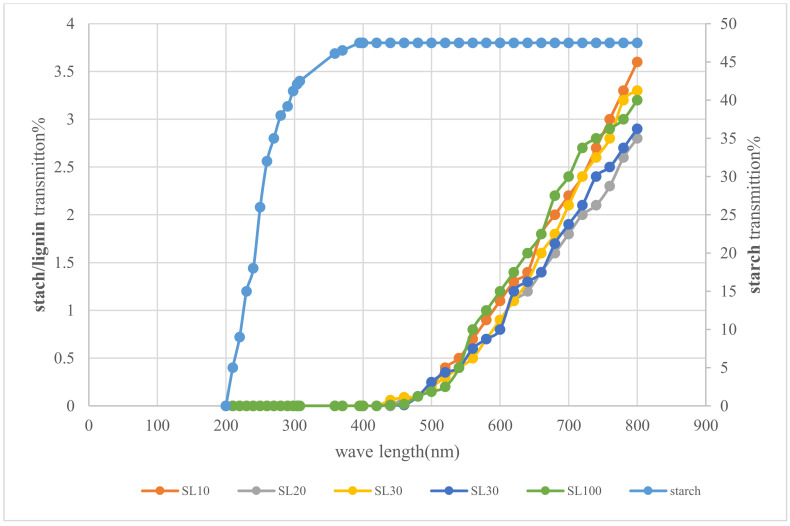
Ultraviolet transmission of neat starch and starch-lignin composite with a different number of grinding cycles.

Lignin from different grinding cycles was used as reinforcement in starch films and the UV protection of the composites was investigated. The transmittance of the lignin-starch film was measured in the wavelength range between 200 and 800 nm, as shown in Figure 7.

The transmittance of pure starch film was high in the entire region. The addition of lignin reduced the transmittance significantly, as the lignin has chromophore functional groups which can absorb a broad spectrum of UV light in range 250–400 nm. Strong absorption was observed in the range 250–400 nm due to the presence of lignin. Chromophores such as phenolic and ketone groups are responsible for UV absorption in lignin structure. However, there was no significant effect on the number of grinding cycles. The result is compatible with the previous studies (Sadeghifar et al. [45] Sirvio et al. [46] and Shankar et al. [14]).

### 3.6. FTIR Analysis

**Figure 8:**
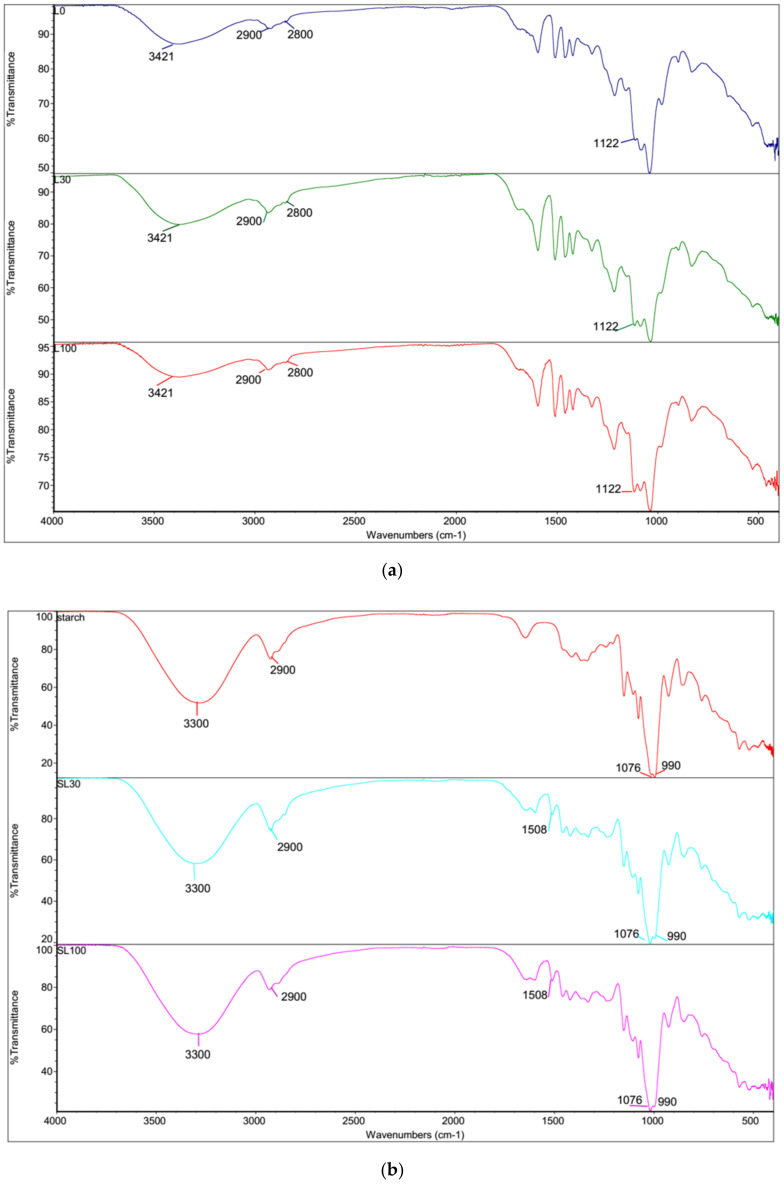
(**a**) Fourier-transform infrared spectroscopy of pure lignin before grinding (L) and after 30 and 100 grinding cycles (L30 and L100, respectively), (**b**) FTIR spectra of starch and lignin-starch composites containing ground lignin from the 30th and 100th grinding cycles (SL30 and SL100, respectively).

The FTIR spectra of lignin before grinding and after each step of grinding are shown in Figure 8a. There were no obvious changes in the spectra in terms of peaks and intensity. Therefore, the number of grinding cycles did not have any effect on lignin structure. However, there was an insignificant change in an absorption peak at 1122 cm^−1^, which is a result of C–H stretching vibration in the aromatic unit [16]. The peak at 3421 cm^−1^ was attributed to the hydroxyl group of phenol and alcohol. The double peaks of lignin at 2900 cm^−1^ and 2800 cm^−1^ are due to stretching of the methyl and methylene groups [41]. Beisl et al. [19], Çalgeris et al. [44], and Zhang et al. [41] presented similar peaks for lignin.

Figure 8b shows the IR spectra of neat starch and lignin starch composites from different grinding cycles. Starch is a polysaccharide while lignin is a polyphenyl-propane polymer with a variety of functional groups. The two most important functional groups in starch are hydroxyl and acetal groups, whereas the lignin has hydroxyl, methoxyl, carbonyl, and carboxyl groups. The peak at 3300 cm^−1^ shows the presence of hydroxyl group of starch, lignin and glycerol. The absorption bands at 2900 cm^−1^ are related to the C–H stretching vibrations in glycerol and starch. The peaks at 1076 and 990 cm^−1^ are characteristics of an hydroglucose ring O–C stretching of starch [44]. After lignin addition, a new peak was recorded at 1508 cm^−1^ that attributed to the aromatic C=C ring deformation [46]. The results are in agreement with previous studies [9,35,46].

In summary, ultrafine friction grinding is a powerful scalable technique for size reduction of lignin and the development of biocomposite materials. Techno-economic analysis is necessary before the process can be scaled up for different applications. Agglomeration of lignin particles is still a challenge that needs to be addressed.

## 4. Conclusions

Lignin was used as a biodegradable filler in the composites. In this work, lignin particle size, which was recovered from bagasse black liquor, was decreased to micro- and nano size via a grinding approach to be more homogeneous and effective in mixing with a starch matrix. The size of the particles can be tailored by adding the grinding steps and more friction in stones during grinding. The obtained lignin was improved in in-homogeneity, which was confirmed by oCelloScope after 100 grinding cycles. Then, composite films containing 10 wt% of lignin from different grinding cycles were prepared by casting method. The transmittance of the starch-lignin film demonstrated the ability of lignin as a UV light blocker. FTIR spectra confirmed the presence of lignin in the composites, but the number of grinding cycles did not change the spectra. This explains that the lignin structure remains unaffected during grinding. Mechanical properties were affected to a large extent by the number of grinding cycles. Even though, a higher number of grinding cycles resulted in smaller lignin particles but the risk of lignin agglomeration during the casting process was increased. The addition of ground lignin increased the tensile strength up to 40% and the elongation decreased by up to 41%.

TGA and DTG curves of starch-lignin film exhibited a different thermal behavior compared to neat starch. Thermal stability of the starch films was improved by the addition of lignin and the grinding cycles affect the stability slightly. SEM micrographs well illustrated the interaction of lignin and starch in the composites. This study provides a chemical free mechanical approach for the development of lignin micro and nanoparticles for being used in starch biocomposites, e.g., for packaging applications.

## Figures and Tables

**Table 1:** Tensile strength and elongation of neat starch and starch-lignin composites with different number of grinding cycles.

	S	SL10	SL20	SL30	SL40	SL100
Tensile Strength (MPa)	3.62 ± 0.2	5.09 ± 0.8	4.45 ± 0.4	4.83 ± 0.1	4.77 ± 0.1	2.62 ± 0.2
Elongation (%)	83.35 ± 0.9	53.56 ± 1.03	65.2 ± 0.4	52.76 ± 0.5	59.13 ± 0.4	48.50 ± 1.06

±indicates the standard deviation.

## Data Availability

The data presented in this study are available on request from the corresponding author.
